# Classical Homocystinuria Incidentally Diagnosed Following a Normal Newborn Screening Result

**DOI:** 10.3390/ijns12030073

**Published:** 2026-09-04

**Authors:** José Manuel González de Aledo-Castillo, Mercedes Casado-Río, Sonia Pajares, Silvia Meavilla Olivas, Laura Gort, Laura Martí Sánchez, Abraham J. Paredes-Fuentes, Leticia Pias-Peleteiro, Marta Gomez-Chiari, Montserrat Quintana Vidaurri, Rosa María López-Galera, Ana Argudo-Ramírez, Jaime Sainz de Medrano, Rafael Artuch Iriberri, Judit García-Villoria, Aída Ormazábal Herrero

**Affiliations:** 1 Division of Inborn Errors of Metabolism-IBC, Department of Biochemistry and Molecular Genetics, CDB, Hospital Clínic de Barcelona, 08036 Barcelona, Spain; spajares@clinic.cat (S.P.); lgort@clinic.cat (L.G.); paredes@clinic.cat (A.J.P.-F.); rmlopez@clinic.cat (R.M.L.-G.); argudo@clinic.cat (A.A.-R.); jisanz@clinic.cat (J.S.d.M.); jugarcia@clinic.cat (J.G.-V.); 2 Fundació de Recerca Clínic Barcelona, Institut d’Investigacions Biomèdiques August Pi i Sunyer (FRCB-IDIBAPS), 08036 Barcelona, Spain; 3 Department of Clinical Biochemistry, Institut de Recerca Sant Joan de Déu, 08950 Esplugues de Llobrega, Spain; mercedes.casado@sjd.es (M.C.-R.); rafael.artuch@sjd.es (R.A.I.); aida.ormazabal@sjd.es (A.O.H.); 4 Centro de Investigación Biomédica en Red de Enfermedades Raras (CIBERER), ISCIII, 28029 Madrid, Spain; 5 Pediatrics, Gastroenterology, Hepatology and Nutrition, Sant Joan de Déu Hospital, 08950 Esplugues de Llobregat, Spain; silviamaria.meavilla@sjd.es; 6 Department of Genetics and Molecular Medicine, Sant Joan de Déu Hospital, 08950 Esplugues de Llobregat, Spain; laura.martis@sjd.es; 7 Neurometabolic Unit, Department of Child Neurology, Sant Joan de Déu Hospital, 08950 Esplugues de Llobregat, Spain; leticia.pias@sjd.es; 8 Clinical Genetics Department, Sant Joan de Déu Hospital, 08950 Esplugues de Llobrega, Spain; mquintana.hj23.ics@gencat.cat; 9 Diagnostic Imaging Department, Sant Joan de Déu Barcelona Children’s Hospital, 08950 Esplugues de Llobrega, Spain; marta.gomez@sjd.es; 10 Institut de Recerca Sant Joan de Déu, 08950 Esplugues de Llobrega, Spain

**Keywords:** classical homocystinuria, cystathionine beta-synthase (CBS) deficiency, newborn screening, false-negative result, pyridoxine responsiveness, total homocysteine, methionine

## Abstract

Classical homocystinuria (HCY) due to cystathionine beta-synthase (CBS) deficiency is included in many newborn screening programs, which traditionally use dried blood spot (DBS) methionine as the primary biomarker. However, some patients have normal or only mildly elevated methionine in the neonatal period, leading to false-negative results. We report a four-month-old male infant referred for sagittal craniosynostosis, whose metabolic evaluation revealed markedly elevated plasma methionine and total homocysteine concentrations. Genetic analysis identified a homozygous, pathogenic variant (c.1007G>A) in the *CBS* gene, confirming HCY. On retrospective review, the DBS sample had been collected at 48 h of life, and methionine was within the reference range. Consequently, the screening algorithm had not triggered second-tier total homocysteine testing. Sanger sequencing of the original DBS sample confirmed the same homozygous *CBS* variant, ruling out sample swap and establishing the case as a true false-negative result. Treatment with pyridoxine and folic acid rapidly normalized biochemical parameters, consistent with a pyridoxine-responsive phenotype. This case illustrates that normal neonatal methionine does not exclude CBS deficiency and that genomic newborn screening approaches could detect this disease more reliably.

## 1. Introduction

**Figure 1:**
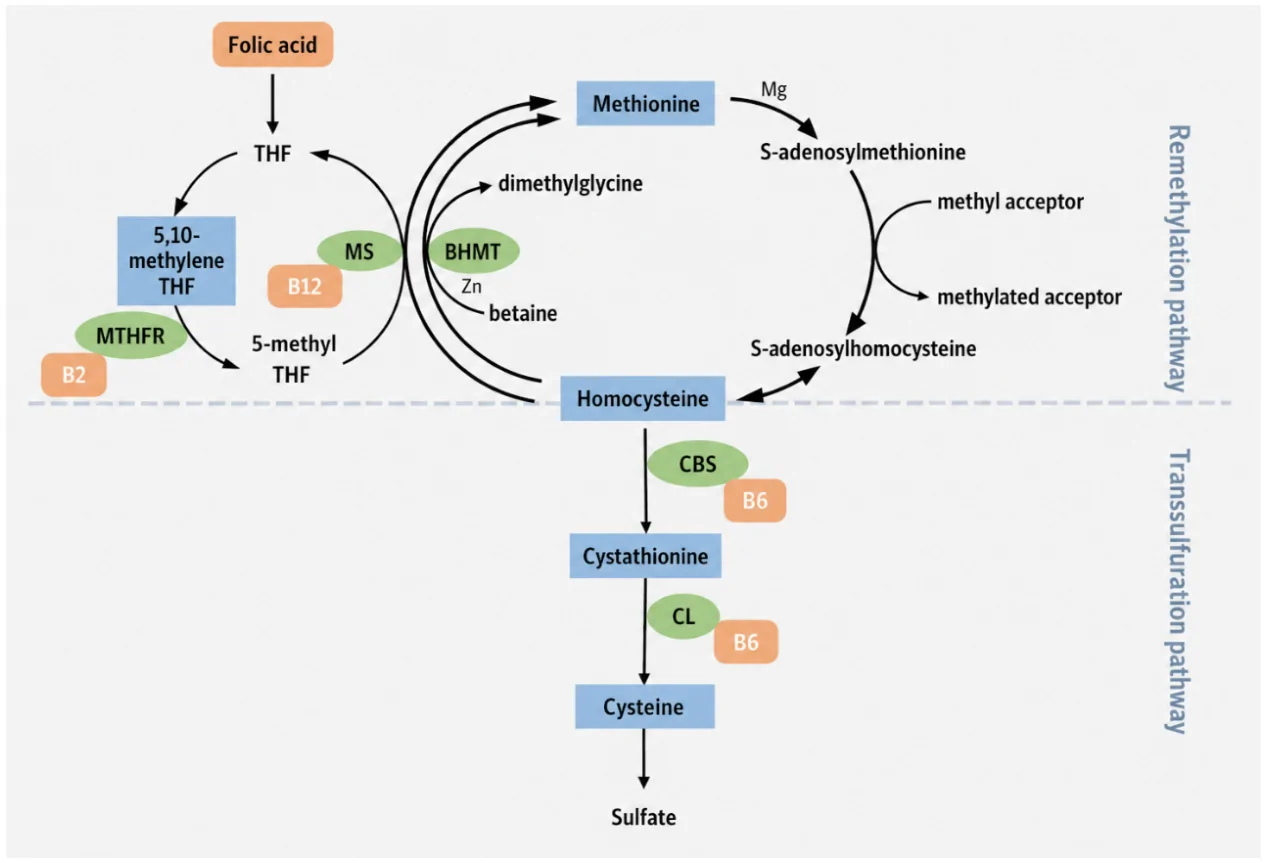
Methionine and Homocysteine Metabolic Pathway. B12: vitamin B12; B2: vitamin B2; B6: vitamin B6; BHMT: betaine–homocysteine methyltransferase; CBS: cystathionine-β-synthase; CL: cystathionine-γ-lyase; Mg: magnesium; MS: methionine synthase; MTHFR: methylenetetrahydrofolate reductase; THF: tetrahydrofolate.

Cystathionine beta-synthase (CBS) deficiency, also known as classical homocystinuria (HCY) (OMIM 236200), is a rare inherited metabolic disorder affecting the transsulfuration pathway of homocysteine, caused by pathogenic variants in the *CBS* gene located on chromosome 21q22.3. This enzymatic defect prevents homocysteine from converting to cystathionine, leading primarily to elevated total homocysteine (tHcy) concentrations and secondarily to increased methionine levels (Figure 1). CBS requires pyridoxal-5′-phosphate, the active form of vitamin B6 (pyridoxine), as a cofactor for proper enzymatic activity [1]. Accordingly, some patients respond well to pyridoxine therapy.

Homocystinuria can also result from genetic defects in the remethylation pathway of homocysteine, including deficiencies of methylenetetrahydrofolate reductase (MTHFR), methionine synthase (MS), and various cobalamin metabolism disorders (e.g., CblE, CblC, CblD, CblF). Acquired forms of homocystinuria can also result from nutritional deficiencies of folic acid or vitamin B12, essential cofactors in the homocysteine remethylation pathway [2]. Recreational nitrous oxide use can also induce functional vitamin B12 deficiency by inhibiting methionine synthase, causing homocysteine to accumulate [3,4]. Although CBS deficiency classically presents with elevated tHcy and variable increases in methionine, other genetic and acquired forms of homocystinuria may show heterogeneous biochemical profiles, sometimes overlapping with HCY [5].

CBS deficiency is clinically classified according to pyridoxine responsiveness as either pyridoxine-responsive or pyridoxine-nonresponsive. Milder variants are more often pyridoxine-responsive, presenting later in childhood or adulthood, with increased residual enzymatic activity, less severe clinical manifestations, and a better prognosis [6]. In contrast, severe pyridoxine-nonresponsive forms typically present in early childhood. Untreated CBS deficiency classically presents with lens subluxation, myopia, intellectual disability, osteoporosis, marfanoid habitus, and thromboembolic complications [7].

Treatment of CBS deficiency primarily aims to reduce tHcy concentrations to prevent or mitigate the clinical consequences of homocysteine toxicity [8]. Pyridoxine-responsive patients are usually treated with oral pyridoxine supplementation. If pyridoxine alone does not normalize biochemical markers, clinicians may add betaine as a methyl donor to remethylate homocysteine to methionine via betaine–homocysteine methyltransferase (BHMT). Nonresponsive or partially responsive patients generally require dietary management: a low-methionine diet that restricts natural protein intake and is supplemented with a methionine-free amino acid formula containing essential and non-essential amino acids, plus carbohydrates, fats, minerals, vitamins, and essential fatty acids [2,9]. Because early treatment prevents most complications, timely diagnosis through newborn screening (NBS) is critical.

NBS for HCY began in the late 1960s in Massachusetts, after Robert Guthrie adapted his bacterial inhibition assay to detect elevated methionine levels suggestive of CBS deficiency. Screening subsequently expanded in the United States and Ireland, regions with a relatively high disease prevalence [6]. Since 2009, CBS deficiency has been included in the U.S. Recommended Uniform Screening Panel (RUSP) [10]. In Europe, NBS coverage of HCY remains inconsistent, with only 19 countries implementing routine screening. Other high-income countries also include HCY in their core NBS panels, among them Canada, Australia, Japan, South Korea, and New Zealand [11]. Spain included HCY in its mandatory national NBS panel for endocrine-metabolic disorders in 2022, with several of its autonomous communities adopting it even earlier [12].

Here, we report a pyridoxine-responsive case of HCY missed by NBS due to normal neonatal methionine levels and discuss the implications for current and future screening strategies.

## 2. Case Presentation

A three-month-old male infant was referred from his regional hospital to a tertiary care center for evaluation of suspected sagittal craniosynostosis, which is a congenital defect in which one or more cranial sutures close prematurely. When this occurs, the skull cannot grow properly, the brain continues to develop, and the head loses its normal, symmetrical shape.

The patient was of Moroccan origin and born to healthy consanguineous parents, who were first cousins. He had one healthy older sister. Pregnancy was uneventful. He was born at 39 + 3 weeks of gestation by spontaneous vaginal delivery. Apgar scores were eight and nine at one and five minutes, respectively. Anthropometric measurements at birth were within the normal range: weight, 3345 g [percentile (P) 50–75]; length, 50 cm (P 50–75); and head circumference, 36 cm (P 75–90; standard deviation (SD) +0.85).

The neonatal period was unremarkable. At two months of age, he was admitted to a regional hospital because of suspected apneic episodes in the context of influenza A infection. During this admission, an abnormal skull shape and an increase in head circumference were noted, with head circumference exceeding the percentile recorded at birth (P > 99; SD +3.7).

He was referred to his regional reference hospital for further evaluation. Physical examination revealed macrocephaly with cranial deformity consistent with scaphocephaly and marked occipital prominence, a very small anterior fontanelle with normal tension, and a bony ridge over the sagittal suture. Additional findings included mild facial dysmorphic features: a broad nasal bridge, anteverted nostrils, hypertelorism, prominent forehead, and low-set, rotated ears. Cranial ultrasound showed enlarged subarachnoid spaces without hydrocephalus. The patient was provisionally diagnosed with craniosynostosis in the context of a possible dysmorphic syndrome and then finally referred to tertiary hospital for further evaluation.

**Figure 2:**
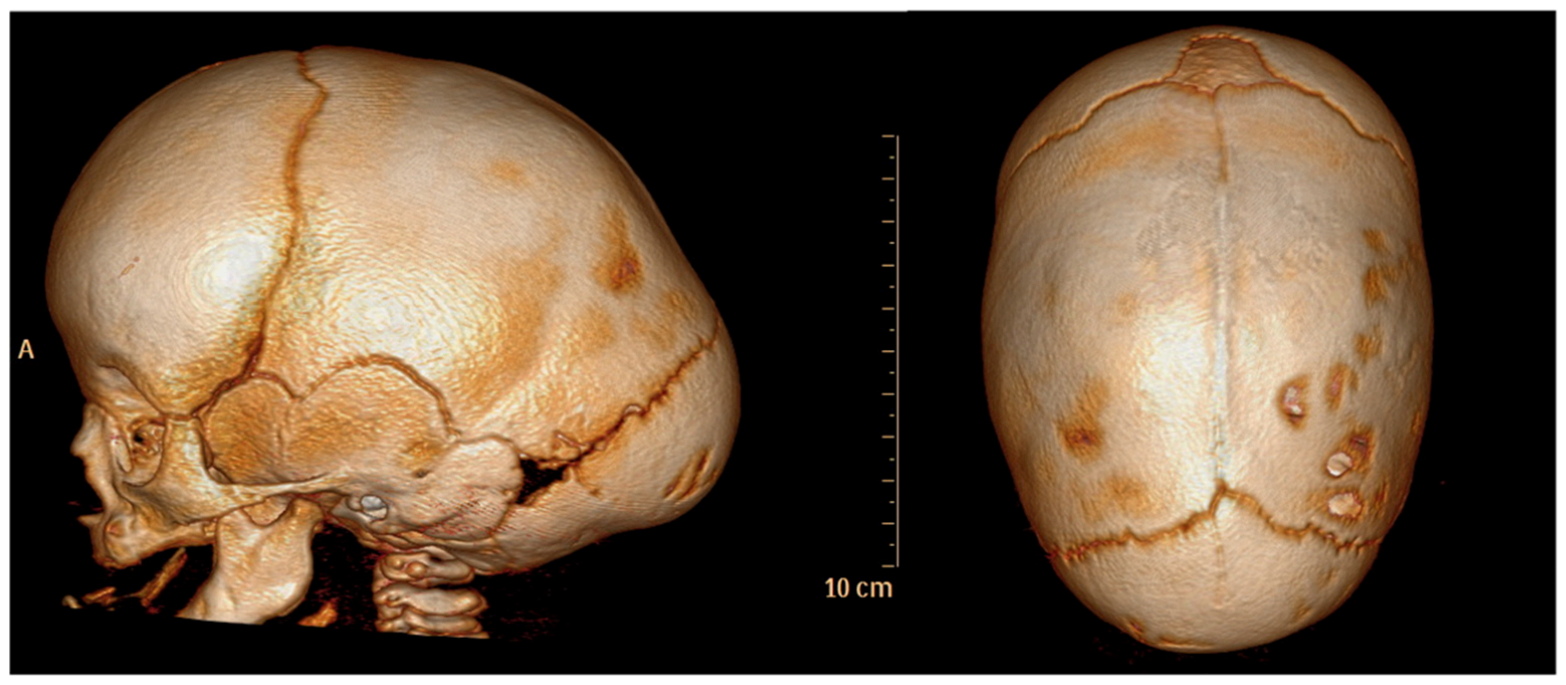
Craniosynostosis on computed tomography. Computed tomography (CT) with 3D reconstruction showing scaphocephaly due to closure of the sagittal suture. (**left**) Lateral view. (**right**) Superior (vertex) view showing the ridge along the fused sagittal suture.

**Figure 3:**
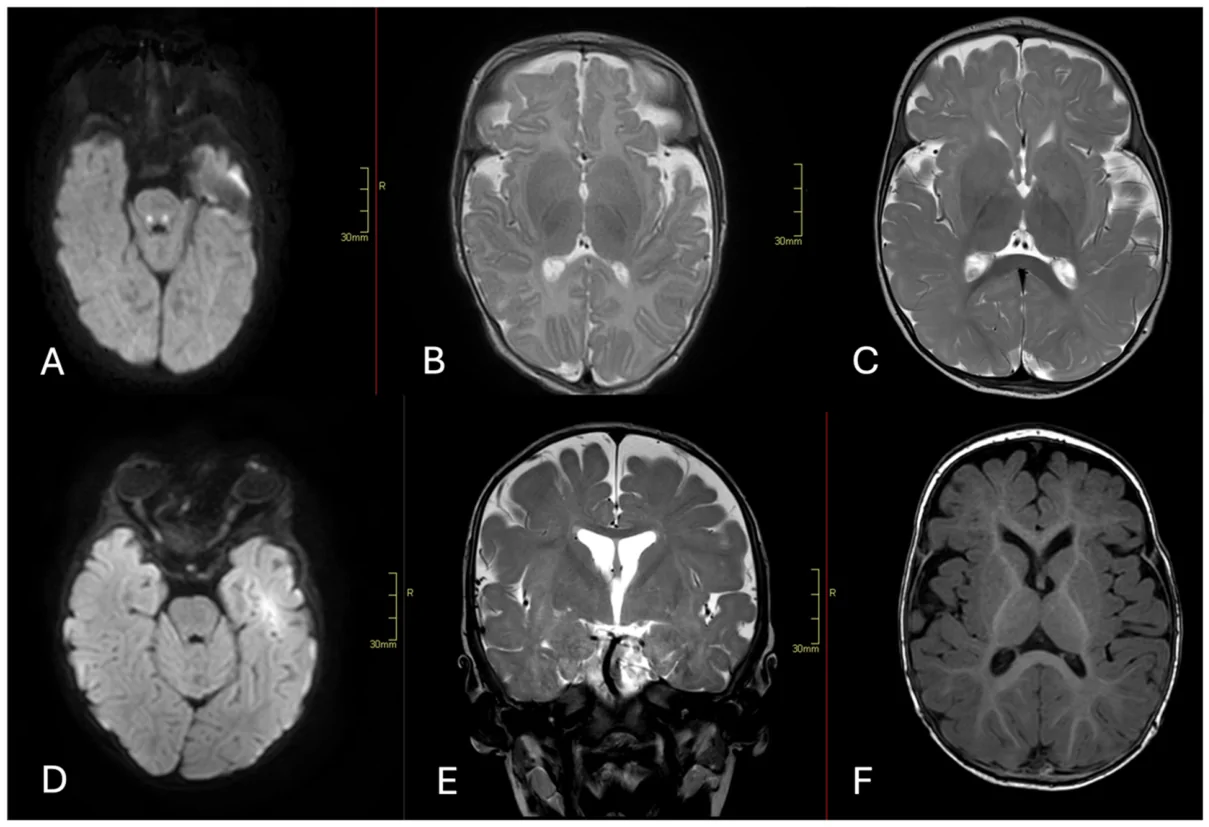
Brain MRI findings at initial presentation and after treatment. (**A**) (MRI study performed at three months of age) Diffusion-weighted imaging (DWI) showing restriction in the dorsal tracts of the brainstem. (**B**) (three months of age) and (**C**) (seven months of age): Axial T2-weighted imaging (T2WI) showing reduction in the size of the thalami and mild T2 hyperintense signal in the globi pallidi. (**D**) (study performed at six months of age): DWI showing resolution of the restriction in the dorsal tracts of the brainstem. (**E**) (six months of age): Coronal T2WI section showing mild T2 hyperintense signal in the globi pallidi. (**F**) (six months of age): Axial T1-weighted imaging (T1WI).

Upon arrival, further neuroimaging studies were performed. Computed tomography (CT) with 3D reconstruction confirmed scaphocephaly due to closure of the sagittal suture, with an associated bony ridge (Figure 2). Brain magnetic resonance imaging (MRI) revealed diffusion restriction in the dorsal tracts of the brainstem, reduced thalamic size, and mild T2-hyperintense signal in the globi pallidi (Figure 3).

A consultation with the Clinical Genetics department confirmed dysmorphic features. Based on the clinical findings and neuroimaging results, a comprehensive laboratory workup was initiated, which included metabolic testing because an underlying metabolic disorder was suspected. General biochemical tests, including blood gas analysis, electrolytes, glucose, creatinine, urea, calcium, lipid profile, transaminases, creatine kinase, and vitamin B12, showed no significant abnormalities. The results of the metabolic studies are summarized in Table 1.

Metabolic testing revealed markedly elevated plasma methionine and tHcy concentrations, suggestive of HCY caused by CBS deficiency.

The hospital contacted the NBS laboratory to discuss the case and review the NBS results. The NBS laboratory confirmed that this patient’s dried blood spot (DBS) sample had been collected at 48 h of life and received at the laboratory five days later. Acylcarnitines and amino acids were within normal ranges. Because the primary marker (methionine: 15.7 μmol/L; reference interval (RI): 6–33) and the Met/Phe ratio (0.4; RI: 0.1–0.68) were both normal, the NBS algorithm did not trigger second-tier tHcy testing. Other biomarkers used to rule out additional diseases in the NBS panel were also normal, and a final normal NBS report was released.

As plasma methionine and tHcy concentrations were markedly elevated on metabolic workup, whole exome sequencing (WES) was performed. This identified a homozygous variant (c.1007G>A) in the *CBS* gene. This variant substitutes arginine for histidine at position 336 of the protein (p.Arg336His) and has previously been described as pathogenic (ClinVar ID 371147) (https://www.ncbi.nlm.nih.gov/clinvar/variation/371147 accessed on 1 May 2026) [13,14], confirming the diagnosis of HCY. In light of the patient’s craniosynostosis and dysmorphic features, the WES panel also included genes associated with craniosynostosis and craniofacial dysmorphism, but no additional conclusive genetic findings were identified. Segregation studies in the parents revealed that both parents were heterozygous for the same variant.

After HCY was genetically confirmed, the NBS laboratory investigated whether a sample swap had occurred. The laboratory considered reanalyzing amino acids in the original DBS sample uninformative because methionine could no longer be reliably measured in the stored sample after three months, likely due to analyte degradation [15]. The laboratory therefore extracted DNA from the original DBS and analyzed the specific c.1007G>A variant identified in whole blood by Sanger sequencing. This study confirmed the homozygous, pathogenic variant (c.1007G>A) in the *CBS* gene, ruling out sample swap. The case was therefore classified as a false-negative result of the NBS.

The patient started on pyridoxine (100 mg/day) and folic acid (10 mg/day), with an excellent biochemical response. Plasma methionine nearly normalized within one week of treatment, decreasing to 38 μmol/L (RI: 12–37 μmol/L), and plasma tHcy decreased to 10.4 μmol/L [RI: <10 μmol/L].

Surgery to correct craniosynostosis was performed without complications. Follow-up MRI at six months of age showed that the diffusion restriction in the dorsal tracts of the brainstem had resolved, with persistent mild T2 hyperintense signal in the globi pallidi (Figure 3).

**Figure 4:**
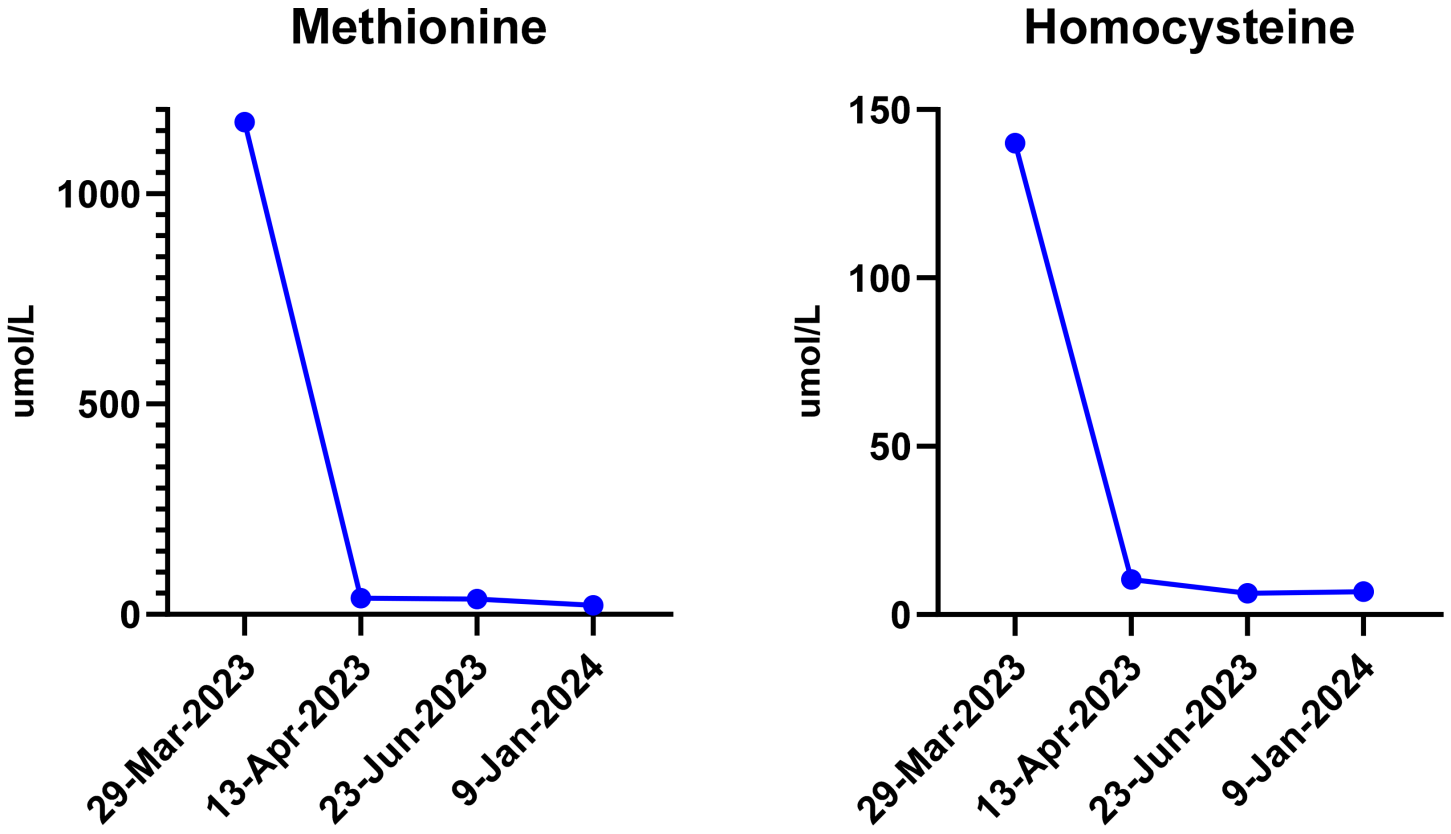
Plasma methionine and total homocysteine (tHcy);), in μmol/L, from initial presentation through follow-up after treatment initiation with pyridoxine and folic acid. Reference ranges: methionine 12–37 μmol/L; tHcy < 10 μmol/L.

At the two-month follow-up, biochemical analysis showed normal methionine (36 μmol/L) and tHcy (6.3 μmol/L) concentrations. At five months after starting treatment, follow-up biochemical testing confirmed that both biomarkers remained within the reference ranges. Figure 4 illustrates how methionine and tHcy evolved after treatment began.

Because patients with HCY are predisposed to lens subluxation, an ophthalmological examination was performed and showed normal, transparent lenses. Annual ophthalmological follow-ups were scheduled to detect potential abnormalities early.

## 3. Discussion

Early diagnosis of CBS deficiency is essential, as treatment initiated during the neonatal period or early infancy can significantly reduce the risk of severe complications [16]. Because CBS deficiency primarily elevates tHcy and, to a lesser extent, methionine, traditional NBS programs have relied on elevated methionine as the primary biomarker for HCY. However, when methionine is used as the sole first-tier marker, sensitivity is limited, particularly in pyridoxine-responsive forms, in which neonatal methionine concentrations may be normal or only mildly increased [17].

Several strategies have been proposed to improve detection. Lowering the methionine cutoff can increase sensitivity, but at the cost of reduced specificity and more false-positive results. Therefore, methionine cutoffs should be defined in relation to local population medians, since methionine measurements vary substantially between laboratories [18]. Adding the Met/Phe ratio, either alone or in combination with methionine, helps detect different forms of CBS deficiency [19]. Similarly, center-adjusted post-analytical tools can further improve screening accuracy [20]. One example is the Collaborative Laboratory Integrated Reports (CLIR) platform, which incorporates covariates such as birth weight, gestational age, and sampling time.

Incorporating tHcy as a second-tier assay in the original DBS sample has been one of the most effective strategies for improving NBS for HCY. When applied following elevated methionine and/or Met/Phe results, second-tier tHcy testing improves both sensitivity and specificity, avoids repeat sampling, and reduces parental anxiety and turnaround time. It has shown clear benefits in programs such as the Catalonian NBS Program [5,21]. However, this strategy still depends on abnormal first-tier markers. The present case would not have been detected by any algorithm in which tHcy measurement is contingent upon elevated methionine or Met/Phe, because methionine was clearly within the reference range in the original neonatal DBS sample. When we reviewed the case and entered the DBS results into CLIR, the resulting score was zero, not informative of HCY.

Although partial pre-analytical degradation of methionine during DBS transport cannot be completely excluded, the magnitude of change required to alter the screening interpretation should be considered. In our patient, the neonatal methionine concentration was 15.7 µmol/L, substantially below our screening cutoff of 33 µmol/L. Therefore, for the original value to have exceeded the cutoff before transport, methionine would have had to decrease by more than 50% during the five-day transport period. Such a decrease is theoretically possible under adverse environmental conditions, as previous short-term stability studies have shown substantial losses of amino acids in DBS exposed to high temperature and humidity, with methionine among the more labile amino acids [22]. However, in the absence of documented extreme transport conditions, this degree of degradation appears unlikely to fully explain the low methionine concentration. Thus, while a minor pre-analytical contribution cannot be ruled out, the result supports the possibility that pyridoxine-responsive CBS deficiency may present with normal or clearly below-cutoff methionine concentrations in the neonatal period.

Another relevant consideration is that some NBS programs include a routine second DBS specimen at one or two weeks of age. Such an approach could potentially increase the likelihood of detecting CBS deficiency in cases with low or only mildly elevated methionine in the first days of life, particularly as methionine concentrations may rise with increasing postnatal age and protein intake [23]. Indeed, previous reports have suggested that a later second specimen may be required to identify some pyridoxine-responsive infants [24]. In our case, a second routine NBS specimen at one or two weeks might therefore have increased the probability of detecting an abnormal methionine or Met/Phe result, although this cannot be stated with certainty given the mild and variable biochemical phenotype of pyridoxine-responsive CBS deficiency. However, requesting a second DBS specimen from all newborns solely to identify the small number of additional HCY cases that may be missed by first-sample screening would substantially increase the complexity, workload, costs, and logistical burden of the NBS program, and may therefore not represent the most efficient strategy in low-prevalence settings.

Despite its diagnostic value, second-tier tHcy testing is not universally implemented, with only 9 out of 24 European laboratories using it [19]. This limited implementation probably reflects the greater analytical complexity of tHcy measurement compared with conventional amino acid and acylcarnitine profiling. Because homocysteine is present in blood mainly in oxidized and protein-bound forms, measurement of total homocysteine requires reduction of disulfide bonds before quantification. Most established approaches rely on LC-MS/MS, which provides robust analytical performance but requires metabolite extraction, chromatographic separation, and specific technical expertise, thereby increasing workload and costs. Nevertheless, tHcy has additional diagnostic value beyond CBS deficiency, as it may also contribute to the detection of remethylation disorders, including severe MTHFR deficiency, methionine synthase and methionine synthase reductase deficiencies, and intracellular cobalamin metabolism disorders such as cblC, cblD, cblE, cblF, cblG and cblJ, some of which may present as methylmalonic acidemia with homocystinuria. In addition, elevated tHcy may identify neonatal vitamin B12 deficiency, often secondary to maternal deficiency [5].

These broader diagnostic implications have supported interest in first-tier tHcy screening. In high-prevalence populations, such as Qatar, first-tier tHcy screening has successfully identified infants with CBS deficiency, remethylation disorders, and vitamin B12 deficiency while reducing recall rates [17,25]. However, applying tHcy measurement to all newborns further increases analytical and operational demands compared with its use as a second-tier assay. More recently, flow-injection MS/MS (FIA-MS/MS) approaches using selective thiol derivatization have been proposed as a way to integrate tHcy into high-throughput first-tier NBS workflows, potentially reducing some barriers by avoiding chromatographic separation and facilitating multiplexing with existing MS/MS assays [26].

False-negative results therefore remain an important limitation of standard NBS for HCY, particularly among pyridoxine-responsive patients, who account for approximately 40–50% of cases [2]. However, false-negative NBS results have also been reported in pyridoxine-nonresponsive CBS deficiency [24,27]. In these patients, cases may be missed because of early sampling, low protein intake, or breastfeeding, any of which can lower methionine concentrations below the screening cut-off. Moreover, the clinical overlap between HCY and other conditions, such as Marfan syndrome, may further delay diagnosis or result in misdiagnosis [28].

The case described in this report highlights a major limitation of NBS for HCY. Pyridoxine-responsive patients may have normal methionine concentrations in the neonatal period and, therefore, may not be detected by screening algorithms that trigger tHcy measurement only for elevated methionine or an increased Met/Phe ratio [16,29]. In this case, craniosynostosis—not a typical manifestation of HCY—prompted the metabolic evaluation and subsequent diagnosis. ClinVar classifies the mutation as pathogenic (ID 371147), and it has previously been described in pyridoxine-responsive HCY patients, though without reported methionine levels [13,30,31].

Our local experience also suggests that pyridoxine-responsive cases may be underrepresented among patients detected by NBS. Over the last 13 years, this is only the second pyridoxine-responsive case identified through our NBS program, whereas four pyridoxine-nonresponsive cases have been diagnosed through the program since 2013. Moreover, we have also identified the same genetic profile in three additional patients with HCY at our center, all of North African origin, who responded excellently to pyridoxine with good metabolic control. These patients were diagnosed clinically because of lens subluxation, without additional major symptoms, and had been born before expanded metabolic screening was implemented in our Autonomous Community. These observations reinforce that patients with normal or only mildly abnormal biochemical profiles during the neonatal period may be missed by current algorithms and diagnosed only later, after ocular, skeletal, vascular, or neurological manifestations appear.

Genetic testing of the *CBS* gene may serve as a complementary strategy to reduce false-negative results. Targeted genetic testing using population-specific *CBS* variant panels has been proposed [32], and genetic screening in Qatar revealed a higher incidence of homocystinuria than biochemical screening alone [17,33]. However, targeted approaches are limited by cost, throughput, and the possibility of missing rare or novel variants not included in the selected panel.

In parallel, pilot genomic NBS programs are underway in the United States, Europe, Australia, and Asia, using exome or whole-genome sequencing during the first days of life [34]. These programs include genes associated with metabolic disorders as well as genes linked to other actionable pediatric conditions [35]. According to a recent publication evaluating gene lists from 27 newborn sequencing programs worldwide, *CBS* is included in 25 of them [36]. To date, only a few programs have published their findings. Although these programs have not yet reported CBS-related HCY cases, they have identified variants in other RUSP-listed metabolic disorders, such as CPT2 and BTD deficiencies, in infants with negative conventional NBS results [37,38].

Genomic NBS remains in an early phase of development, but it is likely to coexist with traditional NBS in the coming years. HCY may represent a paradigmatic example of how both approaches could complement each other. Biochemical screening rapidly and cost-effectively detects many affected newborns and can help interpret variants of uncertain significance, whereas genomic screening may help identify patients with mild or pyridoxine-responsive forms who have normal methionine concentrations at birth. In addition, genomic data may deepen understanding of the mutational spectrum of CBS deficiency, identifying novel pathogenic variants.

However, genomic NBS is only as effective as the gene lists, variant classification rules, filters, and interpretation algorithms applied [39]. Restricting analysis to known pathogenic or likely pathogenic variants may preserve specificity but may also miss rare, novel, deep intronic, structural, or currently variants of uncertain significance (VUS) that are pathogenic in vivo [40]. Conversely, reporting VUS could increase false-positive referrals, parental anxiety, confirmatory testing, and demand for genetic counselling, particularly when no abnormal biochemical phenotype is present to support variant interpretation [41]. Compared with conventional biochemical screening, genomic NBS requires additional pre-analytical processes, DNA extraction, sequencing or targeted library preparation, bioinformatic analysis, variant interpretation, data storage, confirmatory testing, reporting pathways, genetic counselling, and mechanisms for periodic variant reinterpretation. These steps may affect turnaround time and laboratory throughput [37] and would increase costs compared with conventional first-tier biochemical assays [42]. Therefore, at present, genomic NBS should not be viewed as a replacement for biochemical screening, but rather as a complementary approach whose clinical utility, scalability, turnaround time, cost-effectiveness, variant interpretation framework, and integration into existing NBS programs require continued evaluation.

## 4. Conclusions

This case suggests that normal methionine concentrations in the neonatal period do not exclude pyridoxine-responsive CBS deficiency, although a partial contribution of pre-analytical methionine degradation during DBS transport cannot be completely excluded. Screening algorithms that trigger tHcy measurement only for elevated methionine or Met/Phe may therefore miss clinically relevant cases. Although adjusted methionine cutoffs, Met/Phe ratios, CLIR-based tools, and second-tier tHcy testing can improve biochemical screening, false-negative results may still occur in patients with mild biochemical phenotypes. Integrating biochemical and genetic or genomic approaches may therefore offer a more comprehensive strategy to reduce missed cases, enable earlier treatment, and improve long-term outcomes in HCY.

## Figures and Tables

**Table 1:** Results obtained in the metabolic study. Values outside the reference range are marked with an asterisk (*); those with clinical significance are additionally shown in **bold**.

Special Metabolic Study		
Sample Type—Magnitude	Result	Reference Values
Pla—Lactate	1.30 mmol/L	0.77–2.44
Pla—Ammonia	23 μmol/L	13–60
**Pla—Homocysteine**	140 μmol/L *	**<10**
Pla—Amino Acid Profile		
Taurine	27 μmol/L *	30–150
Aspartic Acid	8 μmol/L	2–20
Hydroxyproline	36 μmol/L	12–99
Threonine	149 μmol/L	58–292
Serine	147 μmol/L	92–197
Asparagine	130 μmol/L *	31–120
Glutamic Acid	55 μmol/L	5–80
Glutamine	377 μmol/L	325–676
Proline	411 μmol/L *	90–355
Glycine	173 μmol/L	109–293
Alanine	405 μmol/L	167–439
Citrulline	22 μmol/L	8–40
α-Aminobutyric Acid	17 μmol/L	5–31
Valine	353 μmol/L *	102–294
**Cystine**	10 μmol/L *	**15–59**
**Methionine**	1170 μmol/L *	**12–37**
Isoleucine	82 μmol/L	32–90
Leucine	149 μmol/L	57–155
Tyrosine	98 μmol/L	41–206
Phenylalanine	71 μmol/L *	40–70
Ornithine	91 μmol/L	30–109
Lysine	280 μmol/L *	112–240
Histidine	71 μmol/L	45–104
Pipecolic Acid	2.2 μmol/L	<5.40
Tryptophan	71 μmol/L	30–85
Arginine	63 μmol/L	47–122
Srm—Sialotransferrin (CDG)	**Glycosylation profile without significant alterations**	
Uri—Organic Acid Profile	**Profile without significant alterations. Normal excretion of glutaric and 3-hydroxyglutaric acids.**	
Uri—Sulfites	Negative	

CDG: congenital disorders of glycosylation; Pla: plasma; Srm: serum; Uri: urine.

## Data Availability

The data supporting the findings of this study are not publicly available due to patient privacy and confidentiality restrictions. Relevant data are available from the corresponding author upon reasonable request and with appropriate ethical approvals.

## Abbreviations

The following abbreviations are used in this manuscript:

HCY	Classical homocystinuria
CBS	Cystathionine beta-synthase
DBS	Dried blood spot
tHcy	Total homocysteine
MTHFR	Methylenetetrahydrofolate reductase
MS	Methionine synthase
BHMT	Betaine–homocysteine methyltransferase
NBS	Newborn Screening
RUSP	Recommended Uniform Screening Panel
CT	Computed tomography
MRI	Magnetic resonance imaging
WES	Whole exome sequencing
